# Ultimate Predators: Lionfish Have Evolved to Circumvent Prey Risk Assessment Abilities

**DOI:** 10.1371/journal.pone.0075781

**Published:** 2013-10-16

**Authors:** Oona M. Lönnstedt, Mark I. McCormick

**Affiliations:** Australian Research Council Centre of Excellence for Coral Reef Studies, and School of Marine and Tropical Biology, James Cook University, Townsville, Queensland, Australia; Institute of Marine Research, Norway

## Abstract

Invasive species cause catastrophic alterations to communities worldwide by changing the trophic balance within ecosystems. Ever since their introduction in the mid 1980's common red lionfish, *Pterois volitans*, are having dramatic impacts on the Caribbean ecosystem by displacing native species and disrupting food webs. Introduced lionfish capture prey at extraordinary rates, altering the composition of benthic communities. Here we demonstrate that the extraordinary success of the introduced lionfish lies in its capacity to circumvent prey risk assessment abilities as it is virtually undetectable by prey species in its native range. While experienced prey damselfish, *Chromis viridis*, respond with typical antipredator behaviours when exposed to a common predatory rock cod (*Cephalopholis microprion*) they fail to visibly react to either the scent or visual presentation of the red lionfish, and responded only to the scent (not the visual cue) of a lionfish of a different genus, *Dendrochirus zebra*. Experienced prey also had much higher survival when exposed to the two non-invasive predators compared to *P. volitans*. The cryptic nature of the red lionfish has enabled it to be destructive as a predator and a highly successful invasive species.

## Introduction

Invasive species are recognised as one of the greatest threats to marine biodiversity worldwide [1], [2], and have been found to cause catastrophic alterations to communities by changing the trophic balance within ecosystems [3], [4]. Many of the invasive species that cause the most dramatic effects are predators. Release from their natural enemies and improper anti-predator behaviours by native prey can exacerbate the negative effects of the invasive species [5], [6]. Whether prey will react appropriately to an alien predator depends on the functional similarity and cues of the new predator to ones that are native to the system. This determines the establishment and spread of the invader and the level of impact on the unwitting community. Understanding the underlying aspects of the encounter between a non-native predator and its prey is key to understanding the success and impact of invaders [7]. However, for many non-native predator species the reasons underlying their success are unclear because of the lack of information concerning the mechanisms that underlie their performance in their native communities.

Responding appropriately to predators requires prey to obtain accurate information on the trophic identity and intention of the predator [8]. Innate information can assist in the identification of predators and is most useful when the range of likely predators is small. Learned information augments innate knowledge and many studies have found that prey possess a variety of sophisticated anti-predator mechanisms whereby they can catalogue predators, reinforce memories or de-emphasise (‘forget’) information that is no longer relevant [9]–[12]. Aquatic organisms in particular have been shown to have well developed mechanisms of identifying and assigning appropriate levels of risk to predator cues that operate through the olfactory and visual systems [13]. When damage released skin extract cues are coupled with the smell or sight of a novel predator, the subsequent smell or sight of the predator alone will elicit an antipredator response, through a process known as associative learning [11]. It is unclear whether or how non-native predators manage to circumvent this extremely efficient and rapid learning mechanism.

In the marine environment there are few examples of predator invasions that have been as destructive to the native marine fauna as introduction of the common lionfish, *Pterois volitans*, to the tropical and subtropical east coast of the United States and Caribbean basin. Native to the Indian and Western Pacific Oceans, the lionfish was introduced to Florida in the mid 1980's [14] and has become widespread throughout the Western Atlantic from Florida Keys to Cape Hatteras and throughout the Caribbean basin [15], [16]. The effects of the introduced lionfish are reverberating through the ecosystem, as these hyper-successful nuisance invaders have already altered recruitment patterns, abundance and species composition on many of the invaded reefs [17], [18]. While many aspects of the trophic ecology of the invading populations have recently come under intense scrutiny [19]–[24], little is known of the ecology of the species in its native habitat. It is only by obtaining a detailed understanding of the encounter between the lionfish predator and its native prey that we can better understand why these predators may have become so successful in their novel system.

In this study we examined how experienced and naïve prey individuals (juvenile damselfish, *Chromis viridis*, hereafter *Chromis*) responded to different cues that signify the presence of three different predators. In a series of three experiments we tested whether *Chromis* were able to learn that the chemical cues, visual cues or combined cues of the red lionfish, *P. volitans*, represented a threat. Responses were compared to prey that had been exposed to cues from a common predatory rockcod (*Cephalopholis microprion*) or a lionfish of a different genus (zebra lionfish, *Dendrochirus zebra*). To determine the role learning plays in influencing survival, naïve and experienced *Chromis* were placed together with one of the three predators for 48h and monitored for survival. We show that the predatory success of the red lionfish lies in its capacity to circumvent prey risk assessment abilities as it is virtually undetectable by a common prey species in its native range. The effectiveness of this ability to block innate antipredator responses of prey has most likely contributed to the ecological success of *P. volitans* in invaded regions.

## Results

**Figure 1:**
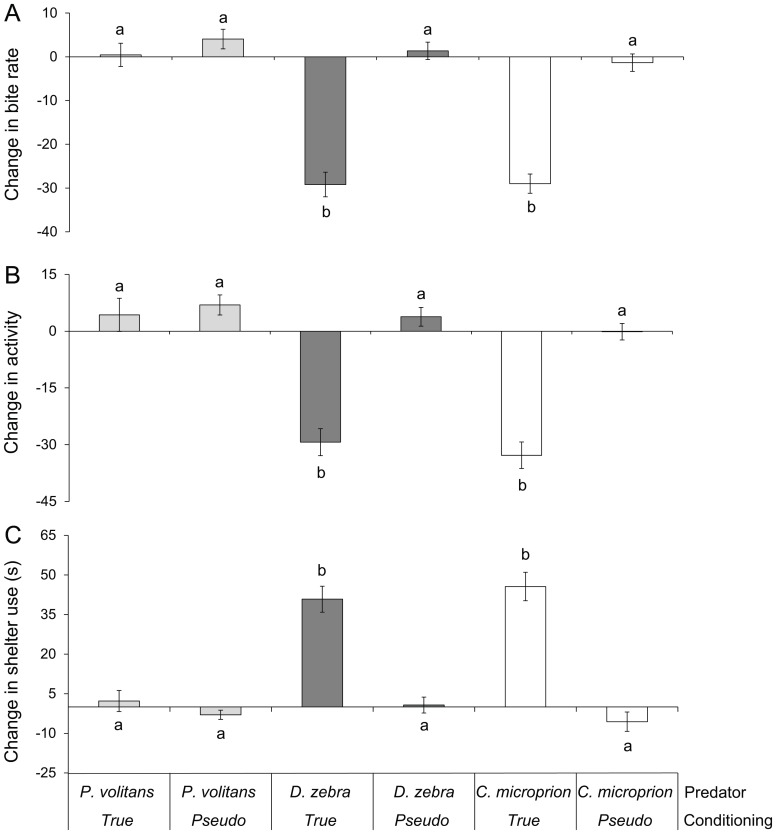
Behavioural responses of inexperienced and experienced juvenile *Chromis viridis* to olfactory cues of three different predators. Experienced prey fed less (A), lowered activity rates (B) and increased shelter use (C) when exposed to olfactory cues of *Dendrochirus zebra* and *Cephalopholis microprion* (N = 16–19). Letters indicate significant groupings.

**Figure 2:**
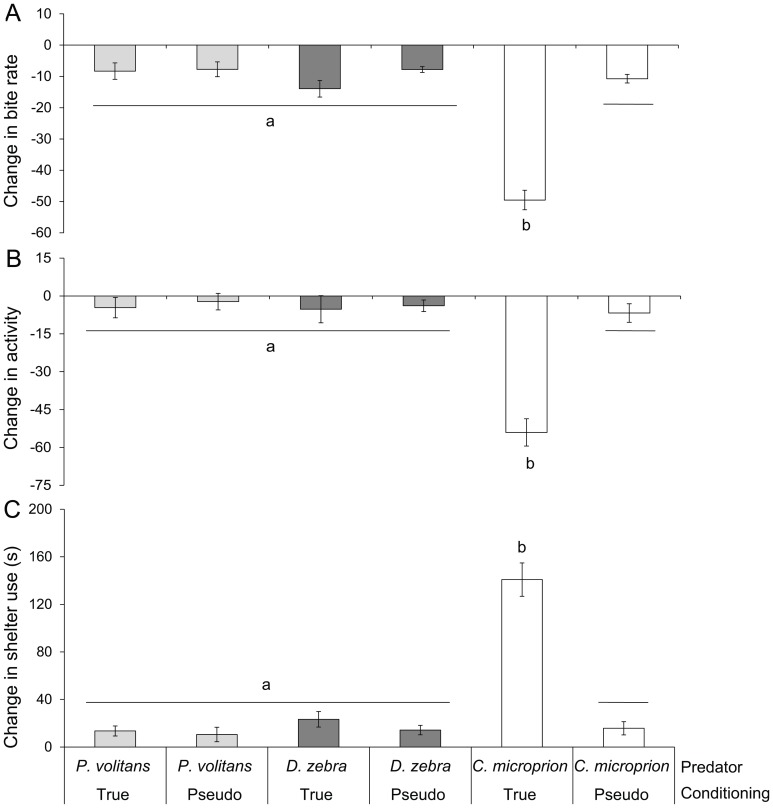
Behavioural responses of inexperienced and experienced juvenile *Chromis viridis* to the visual presentation of three different predators (N = 16–18). Antipredator responses were only seen in experienced prey exposed to *Cephalopholis microprion*. Prey reduced foraging (A), lowered activity rates (B) and increased shelter use (C). Letters indicate significant groupings.

**Figure 3:**
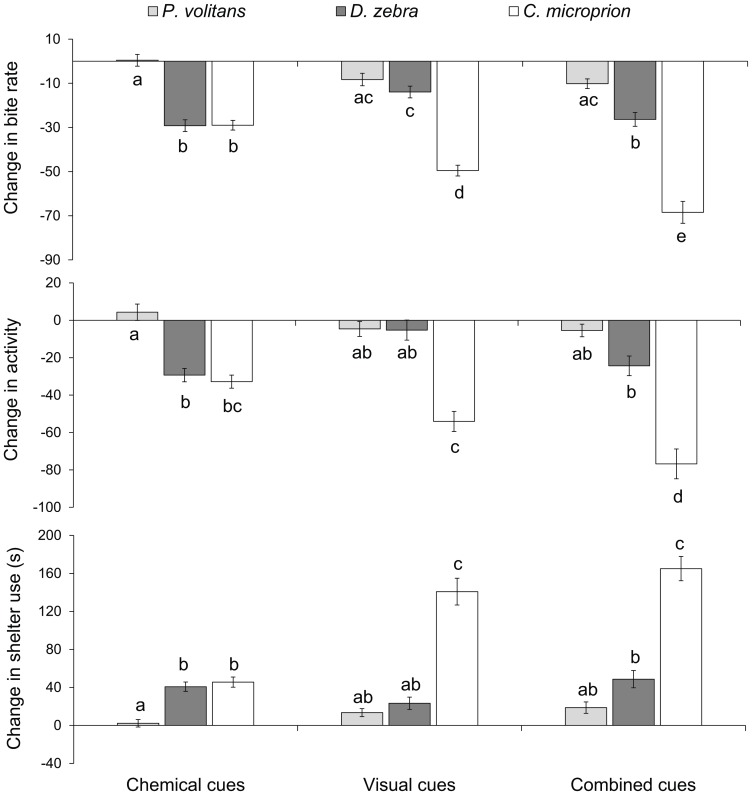
Behavioural responses of experienced juvenile *Chromis viridis* to the exposure of olfactory, visual and a combination of visual and olfactory cues of three different predators (N = 16–19). Prey did not respond with antipredator behaviours when exposed to any threat cues from *Pterois volitans*. Antipredator responses were seen when prey were exposed to olfactory cues of *Dendrochirus zebra*, but not to visual cues alone. When exposed to olfactory and visual threat cues of *Cephalopholis microprion* prey responded with reduced foraging (A), activity (B) and increased shelter use (C), and there was an additive effect when both cue sources were present. Letters indicate significant groupings.

Behavioural responses of *Chromis* to predators differed significantly depending on both type of cue and the species of predator they were exposed to (MANOVA: Olfactory, Pillai's trace_6,174_ = 0.5, *P*<0.0001; Visual, Pillai's trace_6,178_ = 0.6, *P*<0.0001; Combination, Pillai's trace _6,180_ = 0.5, *P*<0.0001). *Chromis* that had been conditioned to learn *Ce. microprion* cues displayed strong anti-predator responses upon presentation of all threat cues associated with this predator, with the strongest responses seen when prey were exposed to chemical and visual cues simultaneously (Figures 1, Figure 2, Figure 3). When exposed to any *Ce. microprion* cue, experienced prey foraged less (MANOVA: Olfactory *F*_2,88_ = 20.4, *P*<0.0001; Visual *F*_2,90_ = 39.4, *P*<0.0001; Combination *F*_2,91_ = 31.9, *P*<0.0001), reduced activity levels (Olfactory *F*_2,88_ = 14.9, *P*<0.0001; Visual *F*_2,90_ = 19.5, *P*<0.0001; Combination *F*_2,91_ = 16.3, *P*<0.0001) and spent more time in shelter (Olfactory *F*_2,88_ = 18.8, *P*<0.0001; Visual *F*_2,90_ = 43.2, *P*<0.0001; Combination *F*_2,91_ = 38, *P*<0.0001) compared with *Chromis* that had no prior experience of the *Ce. microprion* (Figures 1, Figure 2, Figure 3).

*Chromis* with prior experience of *D. zebra* responded to the odour of the predator with reduced activity and feeding, as well as an increase in shelter use compared with inexperienced prey (Tukey's HSD test: *P*<0.0001; Figure 1). There was no response to the visual appearance of *D. zebra* regardless of experience (Tukey's HSD: *P*>0.05; Figure 2). The simultaneous presentation of *D. zebra* scent and visual cue resulted in a similar anti-predator response in experienced prey compared to the response to olfactory and visual cues alone (Tukey's HSD: *P*>0.05; Figure 3). Regardless of experience, there was no response of prey to any predator cue associated with the common lionfish, *P. volitans* (Tukey's HSD: *P*>0.05; Figures 1–3). When exposed to *P. volitans* scent, visual presence or the combination of these cues prey did not appear to visibly react; they continued foraging at a similar rate as pre-exposure.

**Figure 4:**
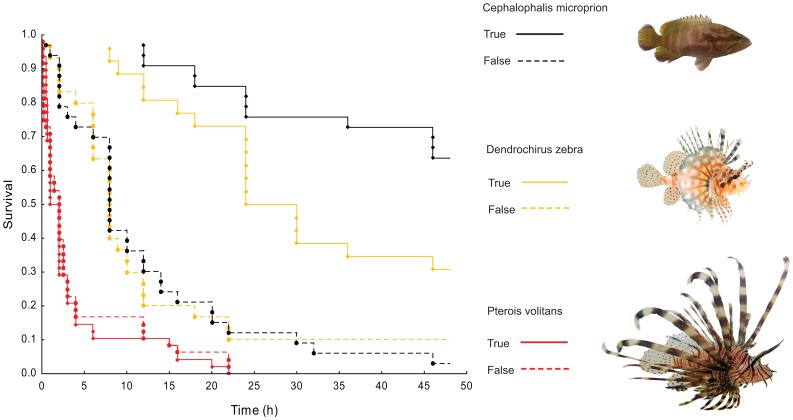
Survival curves (Kaplan–Meier plot) of experienced (true conditioned; exposed to the combination of predator visual presence, odour and conspecific skin extracts) and inexperienced (false conditioned; exposed to the combination of predator visual presence, odour and seawater) *Chromis viridis* to three different predator species (N = 33–49).

Survival trials of *Chromis* revealed a strong influence of both experience and type of predator (Kaplan-Meier survival plot *x^2^*_5_ = 133, *P*<0.0001; Figure 4). Regardless of experience, all prey exposed to *P. volitans* were consumed within 24 hours after release with the majority (true conditioning = 79%, N = 49; false conditioning  = 77%, N = 48) being eaten within the first 3 hours. Experienced prey placed together with *Ce. microprion* had a significantly higher survival with only 33% of individuals being consumed after 48 hours, while 94% of the inexperienced prey were eaten after 48 hours (N = 33). Experienced *Chromis* exposed to *D. zebra* displayed an intermediate survival pattern with 43% uncaught after 24 hours and close to 30% still alive after 48 hours (N = 36) while only 7% of the inexperienced prey (N = 39) remained uncaught after 48 hours.

## Discussion

Our results show that the response of damselfish prey to three different predators greatly differs depending on predator species, threat signal (olfactory, visual or a combination of both) as well as previous experience. Experienced prey will respond strongly to a rockcod threat regardless of the cue, while the physical appearances of the two lionfish species prevented prey from detecting their presence, instead labelling them as non-threatening animals. In fact, irrespective of previous experience, damselfish prey did not respond to any signals, be they visual or chemical cues from the common lionfish, *P. volitans*. Survival patterns of prey emphasized the importance of behavioural responses, as damselfish with previous experience of *C. microprion* had learnt to evade the predator, displaying significantly higher survival rates than inexperienced prey or those exposed to either lionfish species. Prey placed together with *P. volitans* did not survive long regardless of experience, highlighting the efficiency of the highly cryptic nature of the common lionfish. Experienced prey placed together with *D. zebra* displayed intermediate survival patterns, suggesting that at least some prey individuals are able to learn to avoid the predator through olfactory cues alone and/or a combination of olfactory and visual predator cues.

This study demonstrates that *P. volitans* have evolved into highly successful predators, with prey unable to recognize body-shape, coloration or scent of red lionfish in their native ranges. The ecological importance of *P. volitans*' ability to circumvent prey risk assessment can be seen in the successful invasion of this species in the Caribbean. This strategy of preventing prey detection, together with life history characteristics such as high reproductive output, rapid range expansions into many different habitats as well as lack of natural predators and/or parasites, helps explain their extraordinary success in colonising new habitats and in devastating native prey populations [3], [25]. A similar pattern can be seen in another highly successful invader, the ctenophore *Mnemiopsis leidyi*, a planktonic predator that is endemic to Atlantic coasts of North and South America. It has invaded several different regions from the Black Sea in the early 1980s through to the fairly recent invasion in the Baltic and North Sea [26], where it has altered the ecosystems by decimating zooplankton stocks, often followed by trophic cascades [27]. Its ecological success is attributed to its highly efficient feeding technique whereby it generates a hydrodynamically silent current that entrains and transports prey while remaining undetected [26]. *Mnemiopsis leidyi*, like *P. volitans*, is a large, slow swimming predator that greatly benefits from remaining concealed until after encountering prey, allowing them to become hyper-successful nuisance predators in introduced regions. Furthermore, alien predators that are more generalised in their feeding habits can exert keystone effects because of their complex roles in community dynamics. Lionfish prey upon fishes from a variety of functional groups (herbivores, detrivores and small predators alike) as well as numerous invertebrates, so their impact spans multiple trophic levels therefore having particularly widespread and detrimental effects on the communities they invade [20], [28].

Our results illustrate the importance of prey detecting and appropriately responding to predator cues, as the predators responsible for the highest prey removal rates were visually and chemically concealed from prey. The appearance of lionfish differ from most other fish predators in that they have an extravagant body shape characterized by long dorsal spines, greatly expanded pectoral fins, as well as several filamentous appendages above and below their eyes and mouth. Taken together with their disruptive body markings (bright white spots throughout, horizontal stripes on body and vertical stripes on fins), the general outline of lionfish may function to continually confuse and lure prey as they are unable to detect and/ or recognize the lionfish as a predator [29]. In terrestrial carnivores, vertical and horizontal stripes provide camouflage by background matching thus allowing the predator to hunt prey undetected [30]. The lack of prey responses to *P. volitans*' olfactory cues may be due to chemical camouflage, where the predator gives off a scent that labels it as non-threatening. Many terrestrial insects display this type of mimicry, which allows them to enter prey territories undetected [31] or hide from their natural enemies [32]. In many cases the chemically cryptic organisms secrete specific substances that hide their presence either through passive [31] or active mechanisms [33]. A less likely explanation is that *P. volitans* is odourless, having a chemically insignificant profile that allows them to merge with the background environment. Whatever the mechanism of olfactory crypsis, the technique is highly effective at allowing these predators to get very close to their prey. This coupled with the visual crypsis and toxic spines make them a dangerous and skilful predator adept at invading new regions.

While the novel predator-crypsis found in the present study may explain in part why red lionfish are so successful as predators, it does not explain their large population sizes as invasive species in the Caribbean ecosystem [14], [15]. There is very little information on the ecology, behaviour and life history of *P. volitans* in their native range that can assist us in understanding their extraordinary success in invaded regions. This lack of information is partly because these fish are highly cryptic when in low densities, with crepuscular or nocturnal activity patterns, and are therefore difficult to observe [22], [24]. Currently, we can only speculate as to the underlying causes of the rarity of red lionfish in their native distribution. Possible causes include a release from their natural enemies, or environmental and biological conditions that influences their reproductive ecology or larval survival. Like most teleost fishes, lionfish are highly fecund [18], however recruit surveys that are conducted along the Great Barrier Reef hardly ever record red lionfish juveniles. This suggests that population sizes may be constrained by processes that affect some aspect of the early life history from gamete viability and embryo development through to larval growth and survival. As red lionfish continue to invade the Caribbean it is important that invasion and evolutionary ecologists maximize their efforts in understanding lionfish ecology in their native ranges.

Our findings suggest that lionfish are one of the definitive fish predators. Their feeding success is not achieved though speed and surprise, but through a unique form of crypsis that circumvents the well-established mechanism whereby prey fishes learn about their predators e.g., [11]. The generality of these risk assessment mechanism [11], [13] suggests that the results should be broadly applicable to most fish prey species. Further research is warranted on how lionfish achieve this crypsis. Informed management and conservation strategies require a better understanding of how their efficient feeding strategy has promoted invasion through the interrelationship between foraging success and other aspects of their ecology, such as enhanced fecundity and offspring survival.

## Materials and Methods

### Ethics Statement

The research was carried out in accordance with the Australian Code of Practice for the care and use of animals for scientific purposes. This work was conducted with the approval and under the supervision of Lizard Island Research Station and James Cook University ethics guidelines (Permit Number: A1593). All procedures were conducted with care to avoid any pain or suffering in animal subjects.

### Study Species and Sampling

The experimental study was conducted at Lizard Island Research Station (14°40′S, 145°28′E), on the northern Great Barrier Reef, Australia during September-December 2012. The blue-green Chromis, *Chromis viridis* (Pomacentridae), is a site-faithful damselfish that is very common on the shallow reefs of the Indo-Pacific. Juvenile *Chromis* are subject to a variety of resident and transient predators. Individuals (12.7±0.4 mm mean standard length SL±SE) were collected as newly settled juveniles from the reef on SCUBA and maintained (in groups of 20 individuals) in 35 L flow-through aquaria with shelter and fed *Artemia* nauplii twice a day. Common lionfish, *Pterois volitans* (129.4±3.9 mm SL), zebra lionfish, *Dendrochirus zebra* (126.9±3.2 mm SL) and the brown rockcod, *Cephalophalis microprion* (129.8±4.6 mm SL) were collected from the fringing reefs surrounding the island and brought back to the research station. *Cephalophalis microprion* is a common predator along the Great Barrier Reef, often found feeding on juvenile damselfish [34]. *Dendrochirus zebra* is a much less abundant component of the reef community than other small predators, but is nonetheless more common in shallow reef areas than other members of the family Scorpaenidae (28). The least abundant of the three predators is *P. volitans* which is native to the GBR, but rarely seen. All predators were maintained individually in 15 L flow through aquaria and fed juvenile fish of the family Apogoniidae. Other studies have shown that Apogoniids do not have damage-released alarm cues that are responded to by damselfishes [35].

### General Experimental Design

When the epidermis of damselfish is damaged they release a species-specific chemical (a chemical alarm cue) that elicits an antipredator response in conspecifics [8], [13]. When this skin extract cue is coupled with the smell or sight of a novel predator, the subsequent smell or sight of the predator alone will elicit an antipredator response, through a process known as associative learning [11], [36]. Using associative learning *Chromis* were taught to recognize chemical, visual or a combination of chemical and visual cues of three predators. To test the idea that associative learning plays an important role in responding to and subsequently surviving predator encounters half (random allocation) of the *Chromis* juveniles were exposed to the chemical, visual or a combination of visual and chemical threat cues paired with conspecific skin extracts (true conditioning resulting in experienced individuals), while the other half were given the threat cue paired with seawater (false conditioning resulting in inexperienced individuals). The experimental procedure was therefore a two-step process that first involved a conditioning phase where fish were exposed to cues of injured conspecifics (true conditioning) or seawater (pseudo conditioning) paired with those of apredator and second, a testing phase, where fish were exposed to the appropriate cue and had their behaviour assessed. The study was conducted as a series of three experiments.

Following conditioning *Chromis* were placed individually into 15 L aquaria (38×27×24 cm) and allowed to acclimate overnight. The basic tank set up included a 2 cm depth of coral sand and a small piece of healthy live hard coral (*Pocillopora damicornis*) for shelter, while a single air-tube was placed at the other end. A second tube was fixed to the aeration tube and allowed the introduction of *Artemia* food or chemical cues. The air facilitated the distribution of the cues throughout the tank, dye trials showed it took 31.4±0.9 s. Prior to the start of the trial, the water flow was stopped and 5 ml of *Artemia* sp (∼800) nauplii were added to the aquaria to stimulate feeding. The behaviour of a single *Chromis* was recorded for a 4 min pre-stimulus period. Immediately following the pre-stimulus period, a further 5 ml of *Artemia* was added and fish were exposed to the appropriate cue treatment. The behavioural response to experimental treatments was quantified by recording: total number of feeding strikes (successful or otherwise), activity (quantified as the number of times a fish crossed a line on the grid (3×3 cm) suspended over the tank), and total time (s) spent within the branches of the coral shelter. Data were analysed as the difference between the magnitude of behaviours before an experimental stimulus and after exposure to a stimulus (post-pre). Owing to the interdependency of the three behaviours, we analysed the three variables together using a one-way MANOVA, followed by univariate ANOVAs for each behavioural variable. Subsequent Tukey's post hoc tests were performed to assess the differences in behavioural responses between the different treatments.

### Learning to recognize predator cues

Our first experiment investigated the ability of juvenile *Chromis* to learn to respond to predator odour alone following the conditioning phase. *Chromis* were conditioned with 20 ml of the odour of either *P. volitans*, *D. zebra*, or *Ce. microprion*, paired with either 10 ml of seawater (pseudo-conditioning) or 10 ml of conspecific skin extract cues (true conditioning) [36], [37]. Predator odour was obtained by leaving individual fish predators in separate 68-l aerated flow-through plastic holding tanks filled with 30-l of aerated seawater. Two pairs of each predator was placed on staggered alternating cycles of 12 h water flow on and approximately 56 h water flow off, to ensure that predator odour was consistently available for experimental use, and stress was reduced. Following the cessation of water flow for 56 h, predator odour was prepared by drawing up the predator water into a syringe. Predator water was drawn from each predator tank within a pair to avoid intraspecific predator variability effects (a protocol used previously; 37). Skin extracts were prepared following methods of Lönnstedt *et al*. [38]. The following day *Chromis* were exposed to the predator odour that they had been conditioned with on the previous day and their behaviour was assessed.

The second experiment examined how well *Chromis* learned to respond to the visual stimuli of the three different predators (*P. volitans*, *D. zebra* or *Ce. microprion*). Individual predators were placed in clear ziplock bags (20×20 cm) with aerated seawater and placed in 15 L aquaria containing groups of prey fish (2–4 individuals). Bags were large enough to allow the predators to move around freely (and extend their pectoral fins) and they often attempted to strike at prey through the bag. *Chromis* were either pseudo-conditioned with seawater or genuinely conditioned with cues from injured conspecifics to recognize one of the three predators. The next day, fish that had been conditioned in groups were placed individually in aquaria and tested for a response to the exposure of the relevant predator. Predators were placed individually in clear zip-locks bag containing water and a thin layer of gravel (ensuring bags settled on the bottom of the tank) and gently introduced at the end of the tank on the opposite side of the coral shelter [38]. The bag was oriented such that the side of the predator was facing the *Chromis*.

Lastly, we tested responses of *Chromis* to the combination of chemical and visual cues of the three predators. Here, juvenile prey were placed in groups of 2–4 individuals in 15 L tanks and exposed to 20 ml of predator odour and the predator inside of a zip-lock bag paired with either 10 ml of seawater or 10 ml of conspecific skin extract. After conditioning individual *Chromis* were acclimated overnight in experimental aquaria and tested for a response to the simultaneous exposure of the appropriate predator odour and visual stimuli the following day.

### Survival trials of prey

The mortality rates were compared among *Chromis* from the six conditioning treatments [three predators (*P. volitans*, *D. zebra* or *Ce. microprion*) by two conditioning treatments (pseudo and true)]. Following conditioning with the pairing of olfactory and visual cues of the relevant predator, 4–6 randomly chosen individuals from the same conditioning treatment were placed in flow-through mesocosm pools (111 cm diameter, 45 cm high, 368 L). Mesocosms were set up as natural habitats containing a 2-cm deep layer of coral sand substrate, four air-stones, and a 30×30×20 cm coral shelter (hard bushy coral; *Pocillopora damicornis*) in the centre. Sea water was pumped directly from the ocean so it followed natural temperature fluctuations. After one hour a predator (either *P. volitans*, *D. zebra* or *Ce. microprion*), present in a standing acclimation tube since the initiation of the trial, was released into the aquarium and survival of prey fish was monitored every 3 hrs for 48 hrs. Survival (up to 48 h) of fish was compared using multiple-sample survival analysis using a Cox's proportional hazard model (STATISTICA v. 10.0). Survival curves of experienced and inexperienced *Chromis* exposed to the three predators were calculated and plotted using the Kaplan–Meier product–limit method.
